# Heritable, *De Novo* Resistance to Leaf Rust and Other Novel Traits in Selfed Descendants of Wheat Responding to Inoculation with *Wheat Streak Mosaic Virus*

**DOI:** 10.1371/journal.pone.0086307

**Published:** 2014-01-31

**Authors:** Dallas L. Seifers, Steve Haber, Terry J. Martin, Brent D. McCallum

**Affiliations:** 1 Agricultural Research Center-Hays, Kansas State University, Hays, Kansas, United States of America; 2 Cereal Research Centre, Agriculture and Agri-Food Canada, Winnipeg, Manitoba, Canada; Portland State University, United States of America

## Abstract

Stable resistance to infection with *Wheat streak mosaic virus* (WSMV) can be evolved *de novo* in selfing bread wheat lines subjected to cycles of WSMV inoculation and selection of best-performing plants or tillers. To learn whether this phenomenon might be applied to evolve resistance *de novo* to pathogens unrelated to WSMV, we examined the responses to leaf rust of succeeding generations of the rust- and WSMV-susceptible cultivar ‘Lakin’ following WSMV inoculation and derived rust-resistant sublines. After three cycles of the iterative protocol five plants, in contrast to all others, expressed resistance to leaf and stripe rust. A subset of descendant sublines of one of these, ‘R1’, heritably and uniformly expressed the new trait of resistance to leaf rust. Such sublines, into which no genes from a known source of resistance had been introgressed, conferred resistance to progeny of crosses with susceptible parents. The F_1_ populations produced from crosses between, respectively, susceptible and resistant ‘Lakin’ sublines 4-3-3 and 4-12-3 were not all uniform in their response to seedling inoculation with race TDBG. In seedling tests against TDBG and MKPS races the F_2_s from F_1_ populations that were uniformly resistant had 3∶1 ratios of resistant to susceptible individuals but the F_2_s from susceptible F_1_ progenitors were uniformly susceptible. True-breeding lines derived from resistant individuals in F_2_ populations were resistant to natural stripe and leaf rust inoculum in the field, while the ‘Lakin’ progenitor was susceptible. The next generation of six of the ‘Lakin’-derived lines exhibited moderate to strong *de novo* resistance to stem rust races TPMK, QFCS and RKQQ in seedling tests while the ‘Lakin’ progenitor was susceptible. These apparently epigenetic effects in response to virus infection may help researchers fashion a new tool that expands the range of genetic resources already available in adapted germplasm.

## Introduction

When a virus systemically infects a plant, it can induce changes in many of the traits expressed by the host during the interval between inoculation and senescence. If the changes are undesirable, they are considered symptoms of disease induced by viral infection. The sexual production of seed, however, usually serves as a barrier to the vertical transmission of the altered traits.

Most cereal viruses contain positive-sense ssRNA and replicate in the cytoplasm. They are therefore not well suited as vehicles for heritably altering the expression of host traits by integrating viral genes into the host genome. Without transmitting its genes vertically or transforming the host genome, virus infection might nonetheless, by more indirect means, be exploited to induce heritable phenotypic changes in a host of such importance as wheat.

Consistent with a definition that describes changes in genome sequences as ‘genetic mutations’ and changes to the control of expression of unchanged genomic coding sequences as ‘epigenetic’ [1] one can discern two broad approaches by which infection with a cytoplasmic ssRNA virus might nonetheless be exploited to induce the expression of novel host traits.

One approach is to induce the activity of agents, such as transposons or retrotransposons that can alter genome sequences and thus induce genetic mutations. Virus-induced mutations have been reported for maize infected by *Barley stripe mosaic virus* (BSMV) and *Wheat streak mosaic virus* (WSMV). BSMV infection of maize induced several genes expressed in the endosperm to mutate at significantly higher rates in the progeny of virus-infected plants than in those of counterpart healthy controls [2]. Further study showed that, when used as parents in crosses, systemically BSMV-infected plants with homozygous dominant alleles, gave rise in the F_2_ and subsequent progeny generations to phenotypic ratios that deviated significantly from Mendelian expectations [3], [4].

Such observations of the consequences of a virus infection in maize show that, at the very least, the infection can induce transposable elements to move and genes to mutate. As such, virus infection could be considered an aspect of the “genome stress” phenomenon whose role in activating transposable elements was described by McClintock [5]. Since the publication of that seminal work, many forms of stress (including pathogen attack) have been shown to activate retrotransposons in plants. Such activated retrotransposons have, fittingly, been described as stress-induced generators of genomic diversity [6].

Recent years have seen increasingly fruitful exploration of the territory at the frontiers between epigenetics and genetics to bring about stable, desirable changes in gene expression without seeking to alter the underlying genomic coding sequences [1], [7], [8]. In a specific instance arising from our efforts to identify new sources of resistance to WSMV, we discovered genetic resistance to WSMV infection in a single accession of CO960293, an elite winter wheat line with a pedigree of WSMV-susceptible parents [9]. In following up the origin of this welcome genetic resistance, we determined that its pedigree parents – and indeed other accessions of the same line – were all susceptible [9]. If the resistance trait had originated once, *de novo,* from a mutation, or from a heritable change in gene expression in adapted, advanced wheat germplasm, it could conceivably do so again, and in other elite wheat lines. We subsequently carried out experiments of prospective design with sublines derived by selfing from the susceptible doubled haploid spring wheat cultivar ‘McKenzie’ and were indeed able to identify and characterize stable, heritable *de novo* resistance to WSMV in several sublines [10], [11], [12].

In this report we show that infecting ‘Lakin’ wheat with WSMV can induce succeeding progeny generations to express *de novo* the desirable trait of resistance to leaf rust caused by specific races of *Puccinia triticina*, a trait that becomes stable and heritable in a subset of descendant sublines with repeated cycles of selection. We also show that other traits are induced *de novo*, including resistance to stem rust caused by specific races of *Puccinia graminis* and a necrosis factor not previously reported in wheat. Virus infection and attendant host responses might therefore be capable of being developed into a tool for deliberately revealing and exploiting desirable traits not currently expressed in target wheat germplasm.

## Materials and Methods

### Virus Source and Enzyme-linked Immunosorbent Assay (ELISA)

Throughout this study *Wheat streak mosaic virus* (WSMV) was used as the inciting agent to bring about phenotypic changes in wheat. The Sidney 81 isolate was used in Kansas [13], [14] and the Indian Head, Canada (IHC) isolate of WSMV was used for the portion of the work conducted at Winnipeg, Canada [15].

Leaves were collected from field-grown, symptomatic wheat at the Kansas State University Agricultural Research Center-Hays (KSU-ARCH). Because samples were collected from the KSU-ARCH research location, no specific permission was required for obtaining the wheat samples, thus no endangered or protected plant species were involved. The indirect ELISA was performed as described previously [16].

### Leaf-, Stripe- and Stem Rust Isolates, Maintenance and Testing

#### Leaf rust

The Kansas PRTUS 50 culture of race MKPS was used and propagated on seedlings of the wheat cultivar ‘Trego’; the methods of the culture maintenance, inoculation procedures, and classification of progenies have been described for leaf rust [17]. Following inoculation with the PRTUS 50 (MKPS) isolate at the two-leaf stage the plants were held in a mist chamber for 24 hr and then grown in a greenhouse at 21°C +/−6°C with a 16 hr photoperiod of natural light. Seedlings were rated for symptom expression after 14 days.

The 06-1-1 isolate of leaf rust race TDBG was collected in August, 2006 in Manitoba, Canada at a commercial farm site where researchers from Agriculture and Agri-Food Canada are permitted to collect samples for the purpose of conducting annual surveys of rust pathogens. The isolate was increased on seedlings of ‘Thatcher’ spring wheat, collected, vacuum-dried and stored at 4°C. Following inoculation with the 06-1-1 (TDBG) isolate at the two-leaf stage test plants were held in a mist chamber overnight and then grown in a greenhouse at temperatures between 15°C and 25°C with supplemental lighting or in a growth chamber at temperatures between 18°C and 20°C with a 16 hr photoperiod.

#### Stripe rust

The stripe rust seedling tests were conducted at Hays, KS. Wheat seed was planted into 30–×70 -cm metal soil-filled flats. At the two leaf stage, the seedlings were inoculated with urediniopores (fresh spores at approximately 3 mg/ml suspended in Soltrol 170 light oil) of race PST-100 of *Puccinia striiformis* [18]. The inoculated seedlings were placed in a dew chamber at 12°C +/−1°C under dark conditions for 16 hr. The plants were then grown in a greenhouse at 22°C +/−6°C with a 16 hr photoperiod of natural light. Seedlings were rated for infection type after 21 days on a 0–9 scale with 0 representing immune and 9 fully susceptible [19]. PST-100 (collected from an unidentified wheat source near Colby, KS in 2005) was maintained by serial transfer on seedlings of cultivar ‘TAM 107’ at 15°C +/−1°C and a 16 hr photoperiod. Observations of stripe rust in the field were made on field trials of wheat lines that were naturally inoculated with an unknown race (s) of stripe rust.

#### Stem rust

Wheat seedlings were analyzed for stem rust phenotype at Manhattan, Kansas using races TPMK, QFCS, and RKQQ. Following inoculation with isolates of these races the plants were held at 20°C. ‘Lakin’ [20], KS09HW28 and ‘Chinese Spring’ [21] served as susceptible controls, while ‘Arkan’ served as a resistant control. Plants were rated using the scale: Resistant = 0; to 2 and susceptible = 3 to 4. For example,;2-C means a range of infection types from fleck (denoted;) to 2- (small to medium sized uredinia surrounded by necrosis or chlorosis). The minus sign (‘–’) indicates the low side of the range and the plus sign (‘+’) the high side. The ‘C’ denotes extra chlorosis. Overall, the;2-C rating is a moderately resistant reaction.

The W1241 isolate of stem rust race TPMK was collected in August, 1997 in Manitoba, Canada and increased on ‘Little Club’ seedlings, collected, vacuum-dried and stored at 4°C; the isolate was periodically renewed on ‘Little Club’ seedlings. Following inoculation with the W1241 (TPMK) isolate at the two-leaf stage, test plants were held in a mist chamber overnight and then grown in a growth chamber at temperatures between 18°C and 21°C with a 16 hr photoperiod. Reactions were read 12–14 days post inoculation as described above.

### Polyphenol Oxidase (PPO) Analysis of Wheat Seeds

The PPO analysis was similar to that described by Shelton [22] with the following modifications. Seeds were milled on a Udy mill (Udy Manufacturing Corporation, Fort Collins, CO) and 0.245 to 0.255 g of the milled sample was placed in a clean, labeled, clear glass vial (25×95 mm, flat bottom). The vials containing the samples were then cooled in a refrigerator to 4°C for 1 hr. The tyrosine solution [1.25 g of tyrosine (Sigma, T-1145, St. Louis, MO 63178)] was added to 500 ml of distilled water containing 0.090 g of Tween 80) was also maintained at 4°C prior to use. To each sample, 2 ml of tyrosine solution was added and was mixed for 5 seconds using a vortex mixer. Following mixing, the samples were held at room temperature (20–22°C) for 30 to 40 min. The samples were then rated for relative PPO content based on color of the mixture based on a 1 to 9 scale for PPO level, where 1 was colorless (no PPO) and 9 was very black (high PPO). The color of the samples was compared to the color of that for the control cultivars ‘Trego’ (rated a 7) and ‘Lakin’ (rated a 3).

### Virus Infection of ‘Lakin’ Wheat, Selection and Subsequent Advance to the Next Generation of Selected Individuals in Field and Greenhouse

#### First and second growth cycles

On September 28, 2001 breeder’s seed of ‘Lakin’, a wheat cultivar generally susceptible to both stripe and leaf rust [23], was planted at the KSU-ARCH at Hays, KS. The wheat was planted at 50.4 kg ha^−1^ in rows spaced 25 cm apart. In the spring of 2002 we identified and tagged 100 individual plants that had symptoms of infection with WSMV. Leaf tissue of each plant was harvested and analyzed by ELISA for infection by WSMV. One hundred plants diagnosed as positive for the presence of WSMV antigens were individually harvested and threshed on a single-plant basis as were an equal number of uninfected control plants growing close to the WSMV-infected plants.

The harvested ‘Lakin’ seed that had been through one cycle of natural infection with WSMV in the field was planted on November 4, 2002 in metal flats (21–×31–cm) filled with soil in the greenhouse. To subject the population to a second cycle of infection with WSMV, the first and second leaves of the emerged plants were mechanically inoculated 11 days later with the Sidney 81 isolate. These plants were then transplanted to a greenhouse in which they were grown directly in soil. After plants had become established, the heat was shut off to vernalize them for six weeks before returning the greenhouse to 18°C. Leaf tissue of each surviving plant was then assayed by ELISA to confirm systemic infection. These plants were grown to maturity.

#### Third and fourth growth cycles

**Figure 1:**
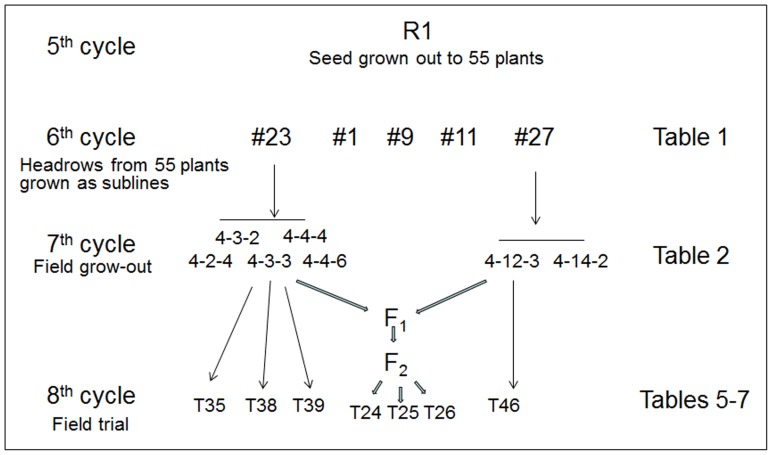
Descent of selected sublines from individual plant ‘R1’. This plant was selected because it expressed stripe and leaf rust resistance *de novo*.

Seed from the 572 surviving individual virus-infected plants was harvested and each plant source kept separate as was the healthy control. This seed was planted in the field in 572 individual head rows 1 m long with 0.305 m row spacing on October 3, 2003 at the KSU-ARCH, Hays, Kansas, as was the seed of healthy controls. During 2004, there was little disease pressure from fungal pathogens and no selections were made. Instead, the seed from 200 of these individual rows was harvested and bulked as was the control. In the fall of 2004, 169 grams of this bulked seed was then planted in a single plot (2 m×60 m, with rows 0.5 m apart) in the field as was the seed from the healthy control in a plot adjacent to it. In the fourth cycle (2004/2005) there was heavy disease pressure from stripe- and leaf rust in the spring and early summer of 2005. Within the stand of wheat plants whose ancestors had experienced pressure from WSMV infection in the first two cycles we were able to identify five individuals (‘R1’through ‘R5’) that were resistant to stripe rust; no such plants were seen in the ‘Lakin’ control. One of these plants (‘R1’) was also resistant to leaf rust. Seeds from these five plants were separately harvested at maturity. A schematic outline of the advance of selected descendant sublines of ‘R1’ in the subsequent fifth through eighth growth cycles is shown in Fig. 1.

#### Fifth cycle

During July of 2005, 34 seeds from the ‘Lakin’ wheat plant ‘R1’ were planted in 4×4-cm soil-filled paper plant bands in the greenhouse. Ten days after planting they were moved to a chamber maintained at 5°C with an 8 hr photoperiod. On Sept 22, 2005 these plants were moved to a greenhouse and transplanted to separate pots. After growing in the greenhouse for six weeks, plants were illuminated with supplemental incandescent light to provide a 14 hr photoperiod, which induced the plants to flower. The remaining seeds of plant ‘R1’ were planted in the field at Hays, KS on Oct. 4, 2005 as five, 1-m long rows. Ten weeks later, 30 emerged individual plants were transplanted to be reared in the greenhouse along with the 34 other plants (described above) that had also been grown from seed of ‘R1’. As some of the transplants from the field did not survive, only 55 plants grew to maturity and produced seed.

#### Sixth cycle

To generate sublines, seed from the 55 selfed plants grown to maturity in the fifth cycle were sown as individual head rows (1-m row, spacing 0.305 m, with 1.5 gram of seed per row) in the field on October 7, 2006. Using a few seeds from each head, a parallel set of 55 sublines was established in pots in the greenhouse and these plants were tested in the spring of 2006 for seedling reaction to inoculation with leaf- and stripe rust.

#### Seventh cycle

In this cycle, there were two parallel tests to evaluate resistance to leaf- and stripe rust of 55 ‘Lakin’-derived sublines: a) an indoor seedling test; and b) a field trial to evaluate adult-plant resistance.

In the indoor trial, sets of 15 to 20 seeds of each of the 55 sublines, descended from ‘R1’, along with the susceptible control ‘Lakin’ were planted into 21×31- cm soil-filled metal flats to test for seedling resistance to leaf- and stripe rust.

In the field trial, sets of 20 seeds of each of the 55 sublines, descended from ‘R1’, along with the susceptible control ‘Lakin’ were seeded in the field in October, 2007. Seed of individual sublines grown out in this cycle became the designated sublines for subsequent detailed analyses and field trials (Fig. 1).

### Leaf Rust Analyses to Determine Inheritance of *de novo* Resistance in Sublines Derived from ‘Lakin’

Individuals sublines descended from ‘R1’ (designated 4-3-3, 2-4-5 and 4-12-3; Fig. 1) were crossed with ‘Morocco’ wheat which was chosen for its susceptibility to leaf rust [24], specifically the MKPS and TDBG races [25]. The F_1_ plants from these crosses were infected at the seedling stage with the 06-1-1 isolate of race TDBG. After ripening, each head from these F_1_ plants was separately labeled and split along the rachis to generate two roughly equal populations of F_2_ seeds corresponding to each individual F_1_ plant; one set of F_2_ plants was then tested at seedling stage using the Kansas PRTUS-50 isolate of race MKPS, the other with the 06-1-1 isolate of race TDBG. Each set of F_2_ plants was seeded randomly within a rectangular array in flats and the number of resistant, mesothetic, and susceptible individuals scored. Mesothetic individuals were counted as susceptible for the purpose of testing hypotheses of inheritance by Chi square analysis [26]. To determine inheritance of *de novo* resistance to leaf rust in a purely ‘Lakin’ background, descendants of the susceptible subline 4-3-3 were crossed with descendants of the resistant subline 4-12-3, and the same protocol followed as that outlined above.

For the analyses at both locations, carried out as described below, the identities of the ‘Lakin’-derived sublines were coded and remained unknown until the analyses were completed. In Kansas, the wheat lines were planted into soil-filled metal flats as described above. The plants were inoculated at the two-leaf stage with urediniospores of the PRTUS-50 (MKPS) isolate. In Manitoba, Canada, seven day old sets of wheat lines were similarly inoculated with the 06-1-1 (TDBG) isolate. The cultivar ‘Morocco’ served as the susceptible check and ‘Fuller’ as the resistant check.

## Results and Discussion

### The Appearance of Altered Phenotypes in ‘Lakin’ Populations Descended from WSMV-infected Ancestors

**Figure 2:**
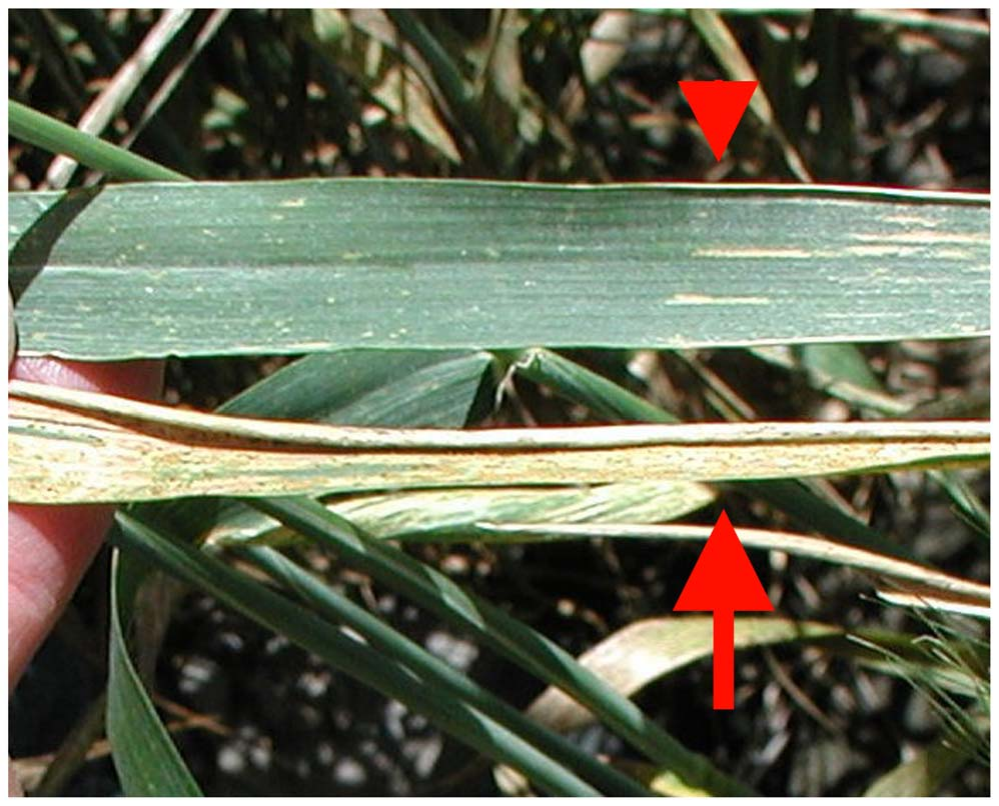
‘Lakin’ wheat in field subject to heavy pressure from natural stripe- and leaf rust inoculum. Arrowhead points down to the greener leaf, from plant designated ‘R1’, which shows resistance to both rusts; arrow points upward to leaf of a typical susceptible ‘Lakin’ plant growing adjacent to plant ‘R1’.

We identified five adult plants (‘R1’ through ‘R5’) that were resistant to stripe rust in the 2004/2005 field experiment (4^th^ cycle, cf. above). This experiment was conducted to compare descendants of ancestors that had been exposed to repeated generations of pressure from WSMV infection with their non-exposed counterparts in a setting where both populations encountered heavy inoculum loads from naturally-occurring stripe- and leaf rust. One of the identified plants, ‘R1’, was also resistant to leaf rust. While plants expressing resistance were observed in the population descended from ancestors grown under pressure from virus infection, all plants of the control population were severely infected by both rusts (Fig. 2).

Seeds from ‘R1’ through ‘R5’ were separately harvested at maturity. Analyses for PPO of subsets of the seeds sampled from all five plants showed they did not differ from the low PPO values of the ‘Lakin’ control’ [23]. Assays by ELISA for infection with WSMV also showed that these five plants (‘R1’–‘R5’) did not contain virus antigens. As an adult plant,’ R1’ had expressed resistance to both stripe- and leaf rust infections, prompting us to investigate: a) how the phenotypes of sublines descended from this plant varied from those of their ‘Lakin’ progenitor; b) if any of the variant phenotypes would come to be stably and uniformly expressed in succeeding generations of descendant sublines; and c) whether the initially *de novo* expression of resistance to stripe- and leaf rust first observed in ‘R1’ could be fixed genetically and then serve as a donor of the trait in crosses with susceptible parents. Three independent lines of evidence point to the variant phenotype(s) arising from changes in expression of genes inherited from the ‘Lakin’ progenitor rather than from accidental outcrossing or seed admixture.

First, the uniformly low level of PPO, characteristic of ‘Lakin’, and observed in ‘R1’ through ‘R5’ and the sublines descended from ‘R1’ is difficult to reconcile with any fortuitous outcrossing, as all of the wheat lines growing near ‘Lakin’ during these studies were lines with the conventional and dominant ‘high-PPO’ trait.

A second trait whose expression in sublines descended from ‘R1’ cannot readily be reconciled with outcrossing is an unusual, apparently *de novo* trait which we describe as ‘progressive necrosis’. This trait has not been reported for any healthy or unstressed wheat line.

### *De novo* Trait of ‘Progressive Necrosis’

Among the 64 plants grown from the seeds of ‘R1’ (5^th^ cycle, cf. above), a subset of five plants developed a novel type of necrosis: as the fourth leaf emerged, the first, or primary leaf, began to develop necrosis, and with the emergence of each new leaf at the growing point the next leaf above the most recently necrotized would in turn develop necrosis and soon die. Despite such necrosis, these plants were able to flower and set seed, but the necrosis did not cease to spread and eventually killed all the leaves prematurely. Seeds from these plants did not fill well but were viable. None of the 64 plants that grew from seeds harvested from ‘R1’ exhibited virus-like symptoms nor (as determined by ELISA) did any contain WSMV antigens, indicating the altered phenotypes were not likely associated with direct effects of virus infection.

**Figure 3:**
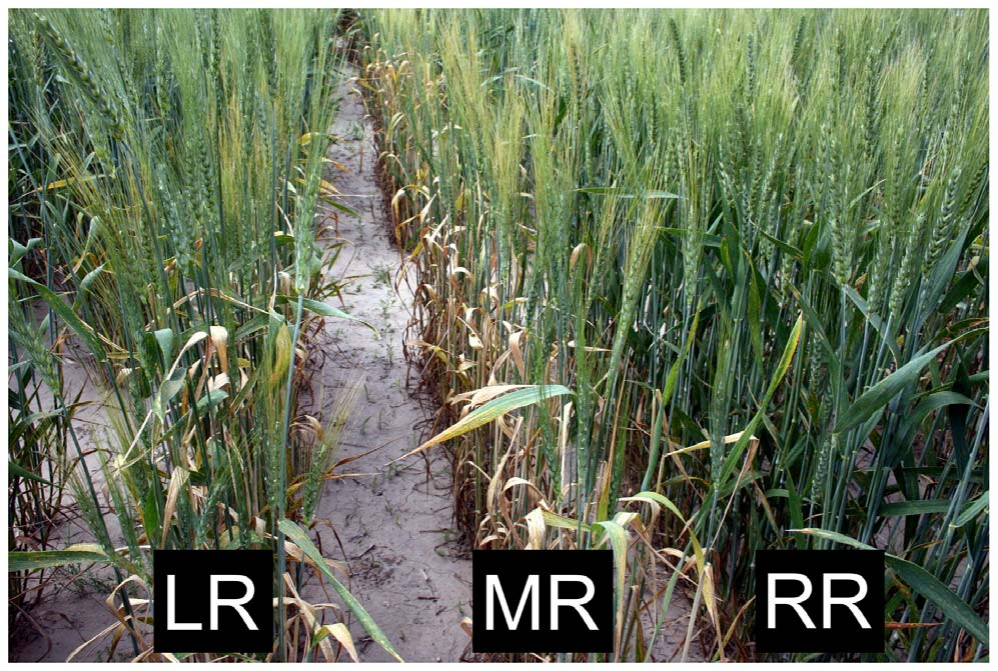
Trait of ‘progressive necrosis’ expressed by ‘Lakin’-derived sublines of the 6^th^ cycle in the 2006/07 field grow-out. Left row (LR) shows a subline comprising plants with and without ‘progressive necrosis’, middle row (MR) a subline uniformly expressing the trait, and the right row (RR) shows the ‘Lakin’ progenitor line uniformly free of ‘progressive necrosis’.

To examine the transgenerational expression of the *de novo* trait of ‘progressive necrosis’, seeds from 55 of the 64 plants of the 5^th^ cycle that set seed were grown out in the 6^th^ cycle as sublines (Table 1; Fig. 3) and the distribution of plants with this trait in each subline recorded. Two of the five 5^th^-cycle individuals displaying progressive necrosis gave rise to sublines that uniformly expressed the trait while the sublines descended from the remaining three contained both necrotic and necrosis-free individuals. Of the 59 plants from the 5^th^ cycle that did not show ‘progressive necrosis’, 51 set seed. Among the 51 resulting sublines only 21 were uniformly free of individuals displaying the trait, while the other 31 contained both necrotic and necrosis-free individuals (Table 1). The trait of ‘progressive necrosis’ was also monitored during all field experiments and not seen in any wheat line to whose pollen the ‘Lakin’ populations and their descendant sublines might conceivably have been exposed. Moreover, two of the three sublines that uniformly express ‘progressive necrosis’ were also tested as seedlings (before necrosis manifests) against leaf rust race TDBG and observed, in contrast to the ‘Lakin’ progenitor, to be uniformly resistant.

**Table 1:** Novel phenotypes of wheat lines arising from the 55 seeds from plant ‘R1’^X^.

		Plant phenotypes^Z^		
Host expression	Lines	Mixed response	Susceptible	Resistant
Leaf rust (field – adult plant)	55	35	12	8
Leaf rust (greenhouse – seedling)	55	35	12	8
*Designations of sublines selected in category*			*#23*	*#1, #9, #11, #27*
Stripe rust (field – adult plant)	51^Y^	22	4	25
Stripe rust (greenhouse – seedling)	55	0	55	0
Necrosis	55	31	3	21

X Observations of altered responses to field (adult-plant) infections with leaf rust, stripe rust and expression of progressive, systemic necrosis under field conditions in the spring of 2007 at Hays, KS.

Y Observations were not recorded for three of the lines for stripe rust reaction because they were susceptible to necrosis.

Z For necrosis, ‘susceptible’ indicates that these plants developed necrosis and ‘resistant’ the absence of necrosis.

### Manifestation of *de novo* Rust Resistance

Finally, outcrossing or seed admixture cannot readily account for the pattern in which *de novo* resistance to leaf rust races MKPS and TDBG manifested among sublines derived from the single plant ‘R1’. There were sublines resistant to both races MKPS and TDBG, others resistant to one but not the other, and still others resistant to neither (Table 2). For outcrossing to account for these outcomes, at least three different wheat plants each with a different race specificity of resistance to leaf rust would have needed to contribute fertilizing pollen.

**Table 2:** Responses to leaf rust (LR) of selected sublines directly descended from the ‘R1’ ‘Lakin’ plant^R^.

		Adult plant^S^	Seedling LR test^S^		
Subline	Tested line (replicated increase)	LR field rating^T^	Resistant	MS^U^	Susceptible
#1	2-4-5	R	5/5 V	0/5	0/5
#1	2-6-2	NR^W^	5/5	0/5	0/5
#9	2-15-1	R	14/15	1/15	0/15
#9	2-15-2	NR	5/5	0/5	0/5
#11	2-19-1	R	0/10	10/10	0/10
#11	2-19-5	R	0/10	10/10	0/10
#23	4-2-4	R	5/10	5/10	0/10
#23	4-3-2	S	5/5	0/5	0/5
#23	4-3-3	S	0/5	4/5	1/5
#23	4-4-4	S	8/9	0/9	1/9
#23	4-4-6	R	14/20	02/20	4/20
#27	4-12-3	R	5/10	4/10	1/10
#27	4-14-2	R	4/10	6/10	0/10
	‘Lakin’^X^	S	0/5	5/5	0/5
	‘Lakin’^X^	S	0/5	5/5	0/5
	‘Lakin’^X^	S	0/5	5/5	0/5
	CO960293	NT^Y^	0/5	0/5	5/5
	‘Morocco’	NT	0/5	0/5	5/5
	93FHB37^Z^	NT	5/5	0/5	0/5

R Adult plants were tested against LR race MKPS in the field at Hays, Kansas and seedling reactions to LR race TDBG were evaluated in greenhouse tests at Winnipeg, Canada.

S Kansas LR isolate PRTUS-50 (typed as race MKPS) and Canadian isolate 06-1-1 (TDBG).

T R = resistant, S = susceptible; rating taken at Hays, KS.

U MS = Mesothetic, responding with both flecks and pustules.

V The numerator indicates the number of plants susceptible to LR and the denominator, the total number of plants inoculated.

W NR = No rating as *de novo* trait of ‘progressive necrosis’ killed tissue before rust inoculation.

X = ‘Lakin’ controls, top to bottom = 1-08-5, 1-07-5, and 2-03-5, respectively.

Y NT = Not tested.

Z 93FHB37 = Winnipeg experimental line used as resistant check.

#### Leaf rust

The resistance to leaf rust that ‘R1’ exhibited as an adult plant in the 2004/05 field trial was expressed by at least some of its progeny. In the 2006/07 field test of the second generation of descendants from ‘R1’ (6^th^ cycle, cf. above; Fig. 1), comprising 55 sub-lines, eight uniformly expressed resistance to race MKPS, 12 were uniformly susceptible and 35 contained both resistant and susceptible individuals i.e., had a mixed response (Table 1). The 55 sublines responded in exactly the same manner in the parallel test of seedling reactions conducted in the greenhouse (Table 1); the same sublines that were uniformly resistant in the field were uniformly resistant in the indoor seedling test, while those that had shown a mixed response did so in the indoor test as well.

#### Stripe rust

The resistance to stripe rust that ‘R1’ exhibited as an adult plant in the 2004/05 field trial was likewise expressed by some of its progeny (Table 1). Of the 51 sublines rated in the 2006/07 field trial, 25 were uniformly resistant as adult plants, 4 uniformly susceptible and the remaining 22 comprised both resistant and susceptible individuals. However, in contrast to the expression of resistance to leaf rust, all 55 sublines were uniformly susceptible as seedlings to infection with stripe rust in greenhouse tests (Table 1).

None of the plants in any of the sublines tested for rust resistance developed virus-like symptoms, nor could we detect virus in these plants by either ELISA or infectivity assay to ‘Tomahawk’ wheat.

### Expression of *de novo* Leaf Rust Resistance in Succeeding Generations of Descendant Sublines

The *de novo* resistance to leaf rust first observed in ‘R1’ was expressed in varying proportions of individuals of descendant sublines (Table 1). Sublines 1, 9, 11, 23 and 27, which were chosen for further analysis (Fig. 1), were examined in replicated tests that were conducted on head-rows sown with the seed of separate individual heads. For example, among the five head-rows of subline 23, two (4-2-4 and 4-4-6) were uniformly resistant in the field trial, whereas three (4-3-2, 4-3-3, and 4-4-4) were susceptible (Table 2, Fig. 1).

The diversity of combinations of phenotypes within and between sublines illustrates the continuing dynamic of changes in trait expression that first became evident in plant ‘R1’ of the 4^th^ cycle (Table 2). For example, plants of head-row 4-3-2 (from subline 23 described in Table 1; Fig. 1) expressed adult plant susceptibility to race MKPS but the subline descended from it in turn uniformly expressed resistance to race TDBG. Conversely, plants of head-row 2-19-1 and 2-19-5 of subline 11 expressed adult plant resistance to race MKPS, while sublines derived in turn from these expressed uniform mesothetic susceptibility to race TDBG. In the case of 2-4-5, resistance to both races was observed and the converse, susceptibility to both races, for 4-3-3. Variation in trait expression could also be seen in the resistance of plants of head-rows 4-2-4, 4-4-6, 2-15-1, 4-12-3 and 4-14-2 to MKPS being accompanied by mixed responses to TDBG in the sublines immediately descended from them. The range of combinations of trait expressions seen in the descendants of ‘R1’ clearly contrasted with the uniform susceptibility to both MKPS and TDBG expressed by three different accessions of the original ‘Lakin’ progenitor (Table 2).

### Utility of the *de novo* Leaf Rust Resistance as a Donor in Crosses: Examination of Inheritance

Seedling tests conducted with race TDBG showed that the *de novo* resistance could be introgressed into susceptible germplasm. In some crosses, the F_1_ population consisted uniformly of resistant individuals, while other crosses yielded both resistant and susceptible individuals (Table 3), an observation that did not conform to what is described as Mendel’s Law of Segregation [27]. Three separate F_1_ populations (A130, A131 and A137) derived from crosses of plants of the susceptible cultivar ‘Morocco’ with three different individuals of the 2-4-5 subline descended from (susceptible) ‘Lakin’ were uniformly resistant. F_1_ populations containing both resistant and susceptible individuals can be seen in the examples (A132 and A133) arising from the crosses of ‘Morocco’ with two separate resistant individuals of the 4-12-3 subline (Table 3). Similarly, crosses made between a susceptible or mesothetic individual of a predominantly susceptible subline like 4-3-3 (Table 2) and a resistant individual of a predominantly resistant subline like 4-12-3 (Table 2) yielded F_1_ populations that were uniformly resistant such as A124 (Table 3) and populations like A122 and A123 that contained at least one susceptible individual (Table 3).

**Table 3:** Inheritance of leaf rust resistance in F_1_ populations in which the resistant parent is an individual from a ‘Lakin’-derived subline expressing *de novo* resistance.

Parents of cross^Y^	Crossidentifier	TotalNumberof F_1_ plants	Response of seedlings in F_1_ population		
			Resistant (R)	Mesothetic^Z^	Susceptible (S)
‘Morocco’(S)×4-3-3 (S)	A136	15	0	0	15
‘Morocco’(S)×2-4-5 (R)	A130	16	16	0	0
‘Morocco’(S)×2-4-5 (R)	A131	16	16	0	0
‘Morocco’(S)×2-4-5 (R)	A137	14	14	0	0
‘Morocco’ (S)×4-12-3 (R)	A132	16	12	0	4
‘Morocco’ (S)×4-12-3 (R)	A133	15	13	0	2
4-3-3 (M)^Z^×4-12-3 (R)	A122	24	23	1	0
4-3-3 (S)×4-12-3 (R)	A123	24	23	1	0
4-3-3 (M)×4-12-3 (R)	A124	24	24	0	0

Y The (S) or (R) following the name of the wheat parent indicates a susceptible or resistant reaction, respectively to the 06-1-1 isolate of LR race TDBG.

Z (M) indicates a mesothetic reaction, one which includes both flecks (resistant reaction) and pustules (susceptible reaction) on the same inoculated leaf.

Certain individual, TDBG-susceptible F_1_ plants (A136.3t1 and A123.2t2) in populations not conforming to the Law of Segregation gave rise to F_2_ populations uniformly susceptible to both TDBG and MKPS races (Table 4). By contrast, two susceptible individual F_1_ plants (A133.5t1 and A133.6t1) in the A133 population gave rise to F_2_ populations uniformly susceptible to TDBG but which had both resistant and susceptible individuals in the portions of the populations inoculated with MKPS (Table 4). In all tests, the susceptible check, ‘Morocco’, was uniformly susceptible and ‘Fuller’, the resistant check uniformly resistant.

**Table 4:** Inheritance of leaf rust (LR) resistance in F_2_ populations^W^.

	Phenotypes^Y^(R:Mesothetic:S)in F_1_ population		Phenotypes (Resistant:Mesothetic:Susceptible)in F_2_ population		
Parents of cross^X^	LR race TDBG	F_1_ source ofF_2_ population	LR raceTDBG	NumberF_2_ seed	LR raceMKPS
‘Morocco’ (S)×4-3-3 (S)	0∶0:15	A136.3t1 (S)	0∶0:15	26	0∶0:11
‘Morocco’ (S)×2-4-5 (R)	16∶0:0	A130.6t1 (R)	12∶1:3	28	7∶1:3 (1n)^Z^
‘Morocco’ (S)×2-4-5 (R)	16∶0:0	A131.1t1 (R)	14∶0:5	33	10∶0:4
‘Morocco’ (S)×2-4-5 (R)	14∶0:0	A137.4t1 (R)	10∶0:4	27	12∶0:1
‘Morocco’(S)×4-12-3 (R)	**12∶0:4**	A132.1t1 (R)	15∶0:7	36	11∶0:1 (2n)
‘Morocco’(S)×4-12-3 (R)	“	A132.2t1 (R)	16∶1:2	33	9∶0:5
‘Morocco’(S)×4-12-3 (R)	“	A132.6t1 (S)	0∶1:9	22	0∶0:12
‘Morocco’(S)×4-12-3 (R)	**13∶0:2**	A133.1t1 (R)	18∶0:7	38	9∶0:4
‘Morocco’(S)×4-12-3 (R)	“	A133.2t1 (R)	16∶0:2	32	8∶0:5 (1n)
‘Morocco’(S)×4-12-3 (R)	“	A133.3t1 (R)	11∶0:2	26	8∶0:5
‘Morocco’(S)×4-12-3 (R)	“	A133.5t1 (S)	0∶0:20	35	**3∶0:11** (1n)
‘Morocco’(S)×4-12-3 (R)	“	A133.6t1 (S)	0∶0:13	26	**2∶0:10** (1n)
4-3-3 (M)^Z^×4-12-3 (R)	**23∶1:0**	A122.1t1 (R)	9∶7:0	30	9∶0:5
4-3-3 (M)×4-12-3 (R)	“	A122.2t1 (R)	13∶0:3	29	8∶0:5
4-3-3 (M)×4-12-3 (R)	“	A122.3t1 (R)	11∶0:3	27	8∶0:5
4-3-3 (M)×4-12-3 (R)	“	A122.4t1 (R)	15∶0:1	30	12∶0:2
4-3-3 (M)×4-12-3 (R)	“	A122.5t1 (R)	15∶1:0	27	8∶0:3
4-3-3 (M)×4-12-3 (R)	“	A122.7t1 (R)	8∶6:0	27	7∶0:6
4-3-3 (M)×4-12-3 (R)	“	A122.8t1 (R)	8∶0:0	15	5∶0:2
4-3-3 (M)×4-12-3 (R)	“	A122.6t1 (M)	0∶2:10	24	0∶0:12
4-3-3 (M)×4-12-3 (R)	“	A122.6t2 (M)	0∶7:9	29	0∶0:13
4-3-3 (S)×4-12-3 (R)	**23∶1:0**	A123.1t1 (R)	20∶1:3	38	12∶0:2
4-3-3 (S)×4-12-3 (R)	“	A123.1t2 (R)	10∶3:1	27	11∶0:2
4-3-3 (S)×4-12-3 (R)	“	A123.1t3 (R)	12∶1:1	25	9∶0:2
4-3-3 (S)×4-12-3 (R)	“	A123.3t1 (R)	13∶0:3	33	12∶0:5
4-3-3 (S)×4-12-3 (R)	“	A123.3t3 (R)	16∶0:4	33	10∶0:3
4-3-3 (S)×4-12-3 (R)	“	A123.5t1 (R)	5∶0:0	10	3∶0:2
4-3-3 (S)×4-12-3 (R)	“	A123.5t2 (R)	16∶3:1	35	10∶0:5
4-3-3 (S)×4-12-3 (R)	“	A123.5t3 (R)	9∶0:0	18	9∶0:0
4-3-3 (S)×4-12-3 (R)	“	A123.6t1 (R)	17∶1:2	36	11∶0:5
4-3-3 (S)×4-12-3 (R)	“	A123.6t2 (R)	13∶0:3	30	8∶0:6
4-3-3 (S)×4-12-3 (R)	“	A123.2t1 (M)	0∶2:12	27	0∶0:13
4-3-3 (S)×4-12-3 (R)	“	A123.2t2 (M)	0∶0:14	28	0∶0:14
4-3-3 (M)×4-12-3 (R)	24∶0:0	A124.1t1 (R)	12∶0:4	30	8∶0:6
4-3-3 (M)×4-12-3 (R)	“	A124.1t2 (R)	13∶2:5	36	10∶0:6
4-3-3 (M)×4-12-3 (R)	“	A124.1t3 (R)	18∶0:1	33	12∶0:2
4-3-3 (M)×4-12-3 (R)	“	A124.2t1 (R)	10∶0:2	25	8∶0:5
4-3-3 (M)×4-12-3 (R)	“	A124.2t2 (R)	11∶2:1	27	9∶0:4
4-3-3 (M)×4-12-3 (R)	“	A124.2t2 (R)	8∶0:2	18	6∶0:2
4-3-3 (M)×4-12-3 (R)	“	A124.5t1 (R)	13∶2:1	31	14∶0:1
4-3-3 (M)×4-12-3 (R)	“	A124.5t2 (R)	15∶1:2	32	11∶0:3

W Tested plants were from single heads of phenotyped individuals of F_1_ populations in which the resistant parent was an individual from a ‘Lakin’-derived subline expressing *de novo* resistance.

X The (S) or (R) following the name of the wheat parent indicates a susceptible or resistant reaction, respectively to the 06-1-1 isolate of LR race TDBG.

Y R = resistant, S = susceptible, and M = mesothetic (both flecks and pustules present on leaves).

Z (n) Denotes plant(s) with progressive necrosis trait; not scored for rust resistance phenotype.

The *de novo* resistance identified in sublines descended from plant ‘R1’ (and therefore from susceptible ‘Lakin’) proved capable of introgression into progeny of crosses with unrelated (‘Morocco’) and cognate (4-3-3, susceptible ‘Lakin’-derived subline) susceptible parents (Table 4).

Where a susceptible individual of a subline derived from ‘R1’was crossed with an unrelated susceptible parent (e.g. ‘Morocco’ x 4-3-3) the F_1_ population was uniformly susceptible to TDBG (Table 4). However, crosses made with resistant individuals derived from ‘R1’ yielded progeny generations in which the expression of the *de novo* trait of resistance to leaf rust appeared in some instances to conform to that of the action of a single, dominant gene, and in others to produce aberrant ratios. Moreover, although all resistant individuals used as trait donors were descended by selfing from ‘R1’, there were progeny populations from crosses that differed in their responses to the TDBG and MKPS races. As expected, F_2_ populations of individuals with resistant reactions to TDBG also had individuals with resistant reactions to MKPS. However, in the A133 F_1_ population arising from the cross ‘Morocco’ x 4-12-3 there were two of 15 individuals susceptible to seedling inoculation with TDBG; the F_2_ populations descended from them in turn were uniformly susceptible to TDBG but encompassed individuals resistant to MKPS or exhibiting the *de novo* trait of ‘progressive necrosis’ (Table 4).

The F_2_ populations (Tables 4,5) descended from F_1_ populations uniformly expressing *de novo* resistance, when tested against TDBG and MKPS produced ratios of resistant to susceptible individuals which were indistinguishable from those expected of the action of a single, dominant gene (Table 5).

**Table 5:** Inheritance of leaf rust (LR) resistance in F_2_ populations descended from F_1_ populations in Table 4 which conform to Law of Segregation.

Parents of cross^X^	Phenotypes^Y^R:M:S in F_1_populationLR race TDBG	Phenotypes (Resistant:Mesothetic:Susceptible) in F_2_ population						
		NumberF_2_ seed	LR raceTDBG	F^2^	P^Z^	LR raceMKPS	F^2^	P
‘Morocco’ (S)×2-4-5 (R)	46∶00:00	98	36∶01:12	0.061	0.804	39∶00:10	0.551	0.457
4-3-3(M)×4-12-3(R)	24∶00:00	232	100∶07:18	1.667	0.196	78∶00:29	0.252	0.615

X The (S) or (R) following the name of the wheat parent indicates a susceptible or resistant reaction, respectively to the 06-1-1 isolate of LR race TDBG.

Y R = resistant, S = susceptible, and M = mesothetic (both flecks and pustules present on leaves); for calculating phenotypic ratios, mesothetic individuals are counted as susceptible.

Z Probability (P) values higher than 0.05 indicate observed ratios do not differ significantly from the 3∶1 ratio of resistant to susceptible individuals of the null hypothesis (H_o_).

If traits evolved *de novo* from the variation induced by epigenetic effects are to be conveniently exploited in crop improvement, they must become uniformly expressed and stably inherited. The next generation of sublines descended from ‘R1’ did not uniformly express *de novo* resistance to leaf rust race TDBG (Table 2), but repeated cycles of selection identified those subsequently descended sublines in which the trait was uniformly expressed and stably inherited (Table 6). For example, while subline 4-12-3 uniformly expressed adult-plant resistance at Hays, KS, the seedling test for resistance to race TDBG identified resistant, mesothetic and susceptible individuals in this subline (Table 2). One of the resistant individuals identified in this test was the progenitor of subline T46 (Fig. 1), which was uniformly resistant in seedling tests against both MKPS and TDBG races and expressed uniform adult-plant resistance in to both leaf and stripe rust in a field trial (Table 6). Other resistant individuals of subline 4-12-3 identified in a seedling test (Table 2) proved effective as donors of the *de novo*-evolved resistance trait in crosses to: a) the unrelated susceptible wheat line, ‘Morocco’; b) the susceptible ‘Lakin’ progenitor; and c) the susceptible ‘Lakin’-derived subline 4-3-3 (Table 3). The inheritance of the *de novo*-evolved resistance trait in the progeny of these crosses (Tables 4a,b) may provide insight into the evolution of a trait arising from epigenetic variation towards a determinant that is capable of further deployment and is apparently fixed genetically.

**Table 6:** *De novo* expression of resistance to leaf rust (LR) and stripe rust (YR)U,V.

		Location of tests								
		2010 Field trial Hays, KS				Hays, KS^W^		Winnipeg, CA^W^		
		LR		YR		LR		LR		
		(MKPS)				(MKPS)		(TDBG)		
Progenitor	Entry	R^X^	S	R	S	R	S	R	M	S
4-3-3	T35	0	8	8	0	0	10	20	0	0
4-3-3	T38	7	0	7	1	11	1	0	19	1
4-3-3	T39	0	7	5	2	0	13	18	0	0
4-12-3	T46	8	0	8	0	15	0	30	0	0
NA^Y^	‘Lakin’	0	11	0	11	0	12	0	20	0
NA	‘Hawken’	7	0	0	7	NT	NT	NT	NT	NT
NA	‘Morocco’	NT^Z^	NT	NT	NT	0	12	0	0	8
NA	‘Fuller’	NT	NT	NT	NT	12	0	NT	NT	NT

U Stripe rust race naturally infecting wheat plants in the field was not determined. For seedling tests, the LR PRTUS-50 source of MKPS and the 06-1-1 source of TDBG were used.

V Tests conducted on eighth-cycle sublines descended from leaf-rust susceptible ‘Lakin’ wheat compared to check cultivars and susceptible ‘Lakin’ progenitor.

W Seedling analyses.

X R = resistant, S = susceptible, and M = mesothetic (both flecks and pustules present on leaves).

Y NA = not applicable.

Z NT = not tested.

The sublines derived from ‘Lakin’ clearly expressed different phenotypes than their progenitor even though no new genes had been introgressed. Uniform *de novo* resistance to both leaf- and stripe rust in the T46 line derived from 4-12-3 is in clear contrast to the non-uniformity of expression of resistance in 4-3-3–derived lines and the uniform susceptibility of the ‘Lakin’ progenitor (Fig. 1; Table 6). Growing repeated generations of ‘Lakin’ wheat under pressure from WSMV inoculation appears instead to have induced some individuals in descendant generations to express *de novo* traits. In turn, some of the sublines descended from these individuals came to express heritably the *de novo* traits in a uniform and stable manner. Thus, the *de novo* resistances to both stripe- and leaf rust that were first observed (at the fourth cycle) in the individual adult plant ‘R1’ become uniformly expressed and inherited traits in a subset of descendant sublines (Table 7) after repeated generations of selection for seedling resistance to leaf rust races MKPS and TDBG.

**Table 7:** Heritability of leaf rust resistance evolved *de novo* in descendant sublines of leaf rust-susceptible ‘Lakin’^V^ in seedling tests to MKPS and TDBG races of leaf rust.

		Leaf rust race^W^ and testing location				
		MKPS – Hays, KS		TDBG – Winnipeg, Canada		
Progenitor	Entry	Resistant	Susceptible	Resistant	Mesothetic^X^	Susceptible
4-3-3	T30	0	10	0	3	9
4-3-3	T31	0	14	0	4	12
4-3-3	T32	0	12	0	12	0
4-3-3	T33	0	10	0	14	0
4-3-3	T34	0	12	0	16	1
4-3-3	T35	0	10	20	0	0
4-3-3	T36	0	8	0	20	0
4-3-3	T37	0	8	0	13	1
4-3-3	T38	11	1	0	19	1
4-3-3	T39	0	13	18	0	0
4-3-3	T40	0	16	0	16	0
4-3-3	T41	0	10	0	12	0
4-3-3	T42	0	13	0	16	0
4-12-3	T43	6	1	6	2	0
4-12-3	T44	10	5	14	1	1
4-12-3	T45	0	12	0	19	1
4-12-3	T46	15	0	30	0	0
NA^Y^	‘Morocco’	0	12	0	0	18
NA	‘Lakin’	0	12	0	20	0
NA	‘Fuller’	12	0	NT^Z^	NT	NT

V Sublines were descended from progenitors that responded to inoculation with *Wheat streak mosaic virus*.

W The LR PRTUS-50 source of MKPS and the 06-1-1 source of TDBG were used.

X Mesothetic (both flecks and pustules present on leaves).

Y NA = not applicable.

Z NT = not tested.

Although the original source of *de novo* rust resistances, ‘R1’, as well as the 4-3-3 and 4-12-3 sublines descended from it uniformly expressed adult plant resistance to stripe rust, this uniformity did not extend to the expression of seedling resistance to leaf rust. In seedling tests against the leaf rust races MKPS and TDBG, sublines in turn descended from 4-3-3 and 4-12-3 were observed that were either uniformly resistant, uniformly susceptible, or comprising both resistant and susceptible individuals (Table 7). For example, in contrast to most sublines descended from 4-3-3, which were uniformly susceptible to both races, subline T35 was uniformly resistant to TDBG but susceptible to MKPS, while subline T39 expressed an opposite profile of resistance to the two races (Table 7).

### Parallel Evolution of Multiple *de novo* Traits Capable of Selection

Taken together, the above observations suggest that the original appearance of *de novo* adult plant stripe rust resistance in plant ‘R1’ was not likely the result of a single mutation which became genetically fixed in its descendants. A prediction that can be made from this perspective is that other, readily testable, *de novo* traits might also be observed in the descendants of individual plants (e.g. ‘R1’), which express altered traits revealed in selection regimes. Although resistance to stem rust was never selected for in any of the cycles leading up to the 2009/10 field experiment, the next generation of six of the sublines also exhibited moderate to strong *de novo* resistance to stem rust races TPMK, QFCS and RKQQ in seedling tests in which the ‘Lakin’ progenitor was, as expected, uniformly susceptible (Fig. 1; Table 8). These observations are not readily explained as the consequences of a single or a small number of specific genetic mutations but, as we shall argue more extensively below, consistent with the hypothesis that our protocol modifies gene expression at several (perhaps numerous) loci simultaneously.

**Table 8:** Phenotypes of wheat plants following inoculation with selected stem rust races.

	Stem rust races^X^		
Wheat^W^	TPMK	QFCS	RKQQ
T24 (4-3-3×4-12-3)F_2_-p1	2+3−Y	22+	;1+
T25 (4-3-3×4-12-3)F_2_-p2	2+3−	22+	;2+
T26 (4-3-3×4-12-3)F_2_-p3	2+3−	22+	;2+
T46 p1	2+3−	22+	;2−
T46 p2	22+	22+	;2
T46 p3	22+	22+	;2-C
‘Lakin’^Z^	33+	34	34
KS09HW28^Z^	33+	3+	34
‘Chinese spring’^Z^	34	34	34
‘Arkan’^D^	;1	1+2−	0;

W T24, T25, T26, T46 are sublines descended from ‘Lakin’ progenitor generations that responded to virus pressure; p1, p2, p3 denote individual plants within sublines as source of seed.

X Seedlings analyzed for stem rust phenotype at Manhattan, Kansas at 20°C.

Y Resistant = 0;2 and susceptible = 34. For example,;2-C means a range of infection types from fleck (denoted;) to 2- (small to medium sized uredinia surrounded by necrosis or chlorosis). The minus (‘–’) denotes the low side of the range and the plus (‘+’) denotes the high side of the range. The C denotes extra chlorosis. Overall, the;2-C rating is a moderately resistant reaction.

Z Controls: ‘Lakin’ [20], Chinese Spring, and KS09HW28 are susceptible in seedling stem rust assays against races while to stem rust races MCCFC, QFCSC, QTHJC, RCRSC, RKQQC, TPMKC, and TTTTF (Data from the 2010 Wheat Regional Germplasm Observation Nursery. Stem rust analysis conducted at USDA ARS, St. Paul, MN by Yue, Jin.). Published on line: (http://arslincoln.unl.edu/wheat), and ‘Chinese Spring’ [21] is the stem rust susceptible control and ‘Arkan’ the resistant control.

The observations discussed here support the case that useful heritable traits such as disease resistance can be evolved *de novo* in plant populations of modest size after only a relatively small number of iterations. While in this study the initial cycles of growing ‘Lakin’ under pressure from WSMV infection involved field-scale population sizes, we have since demonstrated the *de novo* evolution of resistance to leaf rust in sublines derived by similar protocols from ‘Thatcher’ (a susceptible spring wheat cultivar) using small populations in each cycle (never more than 40 plants in any given population) and yielding sublines with uniform resistance after as few as five cycles [Haber, *pers. comm.*] We have also evolved *de novo* resistance to WSMV itself in sublines similarly derived from the virus-susceptible spring wheat cultivar ‘McKenzie’ using small populations and achieving uniform resistance after only four cycles [12].

### Range of Applicability in Elite Wheat Germplasm

If the approach to evolving resistance *de novo* we are describing here is to be of more general utility, it should be capable not only of yielding improved descendant sublines from small populations after modest numbers of cycles, but also be effective on more than a small proportion of the adapted elite germplasm to which it is applied. To show this is feasible, we applied a modified, scaled-down iterative protocol to derive rust-resistant sublines from two Kansas winter wheat cultivars (‘2137’ and ‘RonL’) that were susceptible to natural leaf rust inoculum in Kansas. In a 2010 field test, the selected fourth-cycle sublines derived from ‘2137’ were uniformly resistant, while third-cycle sublines derived from ‘RonL’ were similarly susceptible as their ‘RonL’ progenitor. Follow-up experiments applied seedling tests against specific races to examine the apparent *de novo* rust resistances evolved in sublines descended from ‘2137’ and ‘RonL’ (Table 9). In the seedling test against leaf rust race TDBG, both the ‘2137’ progenitor and the selected subline gave resistant reactions, but the progenitor and evolved descendant sublines were distinguished by their clearly distinct seedling reactions to stem rust race TPMK (Table 9). The pattern was similar for the evolved ‘RonL’ subline descending from KS10HW190-5 (clear expression of seedling resistance to TPMK stem rust) but not seen in the counterpart descending from KS10HW190-6 (Table 9).

**Table 9:** Evolution of *de novo* resistance to *Puccinia* spp. in responses to rust inoculations in sublines descended from susceptible progenitors.

	Stripe Rust	Leaf Rust	Leaf Rust	Stem Rust
	2010 spring	2010 spring	Seedling test	Seedling test
	natural	natural	Race	Race
Line	inoculum	inoculum	TDBG	TPMK
‘2137’ (foundation seed)	S^Z^	S	R (;1+)^Z^	MR(22-) & S (33+)
‘2137’ (KS10HW157-2)	R	R	R (;1+)	R (;1-)
‘2137’ (KS10HW157-2 → B381)	*n/a*	*n/a*	R (;1-)	R (0;)
‘RonL’ (foundation seed)	MR^Z^	S	MR (2-)	S (33-)
‘RonL’ (KS10HW190-5)	MR	S	R (;1-)	R (0;)
‘Ron’L (KS10HW190-5 → B393)	*n/a*	*n/a*	R (;1 = )	R (0;)
‘RonL’ (KS10HW190-6)	MR	S	R (;1-) & MR (22-)	S (34-)
‘RonL’ (KS10HW 190-6 → B394)	*n/a*	*n/a*	R (;1-)	S (34)

Z S = Susceptible, MR = moderately resistant and R is resistant = 0;2 and susceptible = 34. The 0 is a resistant response, the (;) denotes hypersensitive fleck response, 1 or 2 denote small to medium sized uredinia surrounded by necrosis or chlorosis, with a 2 denoting more numerous uredinia, and 3 or 4 denote medium to large uredinia surrounded by large areas of necrosis or chlorosis. The minus (‘–’) denotes the lower end of the range and the plus (‘+’) the higher end of the range.

While a small number of additional cycles of testing and selection may be needed to show that the rust resistance traits thus evolved *de novo* will continue to be uniformly expressed, this is worthwhile if it reveals durable sources of resistance in germplasm hitherto regarded as susceptible. It is striking how rapidly and effectively the protocols described here have brought about the evolution of desirable *de novo* traits and their fixation as heritable and genetically useful entities.

### Epigenesis and the Apparent Evolution of Traits *de novo*

We can only speculate right now as to the mechanism(s) that drive the evolution of the specific *de novo* phenotypic changes that we observed in the ‘Lakin’ ‘R1’ plant and the sublines descended from it. However, exploring a range of hypotheses that fit the findings of this study and the growing body of literature describing the heritable, altered expression of plant genes could help point the way to experiments that test explanatory models and expand the scope of applications.

The simple hypothesis that sequences derived from the virus genome became integrated into host DNA is unlikely as WSMV replicates in the cytoplasm; to date, only plant viruses with DNA genomes or those which have a DNA phase during replication have been shown to integrate portions of their sequences into plant host genomes [28]. Moreover, while the progeny of maize plants that had experienced WSMV infection displayed frequent mutations in subsequent F_1_ and F_2_ generations [29], no sequence of the WSMV genome could be identified in affected plants [30].

Similarities in the pattern of emergence *de novo* of novel traits in our study with the expression in subsequent generations of systemic acquired resistance (SAR) [1], [8] suggest that WSMV might more likely exert its apparently epigenetic effects – with subsequent genetic fixation – via the effects exerted by ‘short interfering (silencing) RNA’ (siRNA) that the host produces in response to infection with an RNA virus [31]. As argued in the discussion of recent work demonstrating how SAR could become expressed in next- and subsequent generations of *Arabidopsis* responding to infection with *Pseudomonas syringae* pv *tomato*, siRNAs can control gene transcription through changes in DNA methylation which, in turn, can affect gene expression [8]. The *de novo* development of resistance to a plant pathogen arising from changes in genomic methylation has been demonstrated convincingly. Akimoto [32] treated rice seeds with the methylation inhibitor 5-azadeoxycytidine, a treatment that was lethal to the majority of seedlings. However, two lines descended from surviving individuals showed altered and heritable phenotypes. One phenotype was dwarfism and the other was *de novo* resistance to *Xanthomonas oryzae*. The resistance to *X. oryzae* was associated with an Xa21G-like protein and it was shown that all cytosines were demethylated in the promoter region of the gene encoding this protein. Expression of Xa21 was not detectable in the wild type where all cytosines were methylated within the promoter region.

It is tempting to speculate that the *de novo* resistance to leaf rust in the ‘Lakin’ ‘R1’ plants might also reflect methylation-related epigenetic changes occurring as a host response to virus infection. Substantial changes in the methylation of the plant genome have been demonstrated in the progeny of tobacco infected with *Tobacco mosaic virus* (TMV) [33]. Using methylation analysis of specific loci, Boyko [33] went on to show profound hypomethylation in several R genes (pathogen resistance gene-containing loci), substantial hypermethylation of actin loci, and no change in methylation at loci of repetitive elements. Similarly, Wada [34] showed that the stress-responsive genes of progeny of tobacco plants that had been infected with TMV were hypomethylated and up-regulated. In this context, it is intriguing that *Pea seed-borne mosaic virus*, an RNA virus that, like WSMV, replicates in the cytoplasm, has been demonstrated to direct methylation of DNA in pea plants [35], [36].

The ability to evolve in just a few generations the stable and heritable expression of altered traits from small populations of identical genotypes suggests that the genetic fixation of novel traits that are epigenetically induced by altered methylation might constitute part of an extended array of adaptation mechanisms accumulated by plant evolution. Hauben et al. [7], succeeded in deriving sublines of isogenic, doubled-haploid self-fertilized canola (*Brassica napus*) with distinct physiological and agronomic characteristics, including increased yield potential, after just four rounds of selection. The genetically identical but epigenetically distinct sublines had physiological characteristics and DNA methylation patterns that were altered from those of their progenitor in a stably heritable manner. In another example, sublines derived by selfing from a single seed of the doubled-haploid wheat cultivar, ‘McKenzie’, stably expressed resistance to WSMV *de novo* after just four cycles of selection from progenitors responding to inoculation with the virus [12].

## Conclusions

We have shown here that new phenotypes could be evolved *de novo* among the descendants of progeny of ‘Lakin’ wheat plants that have responded to infection of progenitor plants with WSMV. We have also strengthened the case that this is but an example of a much more general phenomenon by deliberately and prospectively evolving resistance to leaf rust *de novo* from small, well-controlled numbers of individual plants of the cultivars ‘RonL’ and ‘2137’ responding to repeated cycles of inoculation with WSMV.

In deliberately applying virus infection to wheat as a tool to evolve heritable traits *de novo,* we identified an unusual trait of ‘progressive necrosis’, along with the more desirable trait of resistance to leaf rust, and in subsequent rounds of applying the protocol, resistance to WSMV as well. Once demonstrated in experiments starting with large (field-plot size) numbers of plants, this evolution of *de novo* resistance to leaf rust could be shown deliberately and prospectively in several other lines of elite wheat germplasm, indicating its reproducibility and wider utility. These findings and similar reports [12] of success in evolving traits *de novo* suggest that the wheat genome already contains coding information for many useful traits that might not currently be expressed or expressed in sufficiently effective coordination. Evolution fashions improved adaptation from the raw material furnished by heritable variation. Plant hosts responding to virus infection may constitute a new source of such variation that we might exploit where our observations permit us to identify the individuals expressing the traits of interest.
